# Health systems readiness and management of febrile outpatients under low malaria transmission in Vanuatu

**DOI:** 10.1186/s12936-015-1017-4

**Published:** 2015-12-02

**Authors:** Dejan Zurovac, Jean-Olivier Guintran, Wesley Donald, Esau Naket, Josephine Malinga, George Taleo

**Affiliations:** Department of Public Health Research, KEMRI-Wellcome Trust Research Programme, Nairobi, Kenya; Nuffield Department of Clinical Medicine, Centre for Tropical Medicine and Global Health, University of Oxford, Oxford, UK; Center for Global Health and Development, Boston University School of Public Health, Boston, MA USA; Malaria, Other Vector Borne and Parasitic Diseases Programme, World Health Organization, Port Vila, Vanuatu; National Vector Borne Disease Control Programme, Ministry of Health, Port Vila, Vanuatu

**Keywords:** Health system readiness, Malaria test and treat policy, Adherence to guidelines

## Abstract

**Background:**

Vanuatu, an archipelago country in Western Pacific harbouring low *Plasmodium falciparum* and *Plasmodium vivax* malaria transmission, has been implementing a malaria case management policy, recommending parasitological testing of patients with fever and anti-malarial treatment for test-positive only patients. A health facility survey to evaluate the health systems readiness to implement the policy and the quality of outpatient management for patients with fever was undertaken.

**Methods:**

A cross-sectional, cluster sample survey, using a range of quality-of-care methods, included all health centres and hospitals in Vanuatu. The main outcome measures were coverage of health facilities and health workers with commodities and support interventions, adherence to test and treatment recommendations, and factors influencing malaria testing.

**Results:**

The survey was undertaken in 2014 during the low malaria season and included 41 health facilities, 67 health workers and 226 outpatient consultations for patients with fever. All facilities had capacity for parasitological diagnosis, 95.1 % stocked artemether-lumefantrine and 63.6 % primaquine. The coverage of health workers with support interventions ranged from 50 to 70 %. Health workers’ knowledge was high only regarding treatment policy for uncomplicated *P. falciparum* malaria (83.4 %). History taking and clinical examination practices were sub-optimal. Some 35.0 % (95 % CI 23.4–48.6) of patients with fever were tested for malaria, of which all results were negative and only one patient received anti-malarial treatment. Testing was significantly higher for patients age 5 years and older (OR = 2.33; 95 % CI 1.48–5.02), seen by less qualified health workers (OR = 2.73; 95 % CI 1.48–5.02), health workers who received malaria case management training (OR = 2.39; 95 % CI 1.28–4.47) and patients with increased temperature (OR = 2.56; 95 % CI 1.17–5.57), main complaint of fever (OR = 5.82; 95 % CI 1.26–26.87) and without runny nose (OR = 3.75; 95 % CI 1.36–10.34). Antibiotic use was very high (77.4 %) with sub-optimal dispensing and counselling practices.

**Conclusions:**

Health facility and health worker readiness to implement policy is higher for falciparum than vivax malaria. Clinical and malaria testing practices are sub-optimal, however adherence to test negative results is nearly universal. Use of antibiotics is irrational. Quantitative and qualitative improvements of ongoing interventions are needed to re-inforce clinical practices in this area characterized by difficult access, human resource shortages but aspiring towards malaria elimination.

## Background

Vanuatu is an archipelago country in Western Pacific harbouring low and declining falciparum and vivax malaria transmission [1]. In 2011, the latest, and the only nationally representative, survey suggested household malaria prevalence of about 1 % [2]. Case management based on universal parasitological confirmation of suspected malaria patients prior to treatment with artemisinin-based combination therapy (ACT), also known as ‘test and treat’ policy, is one of the key malaria control strategies in the country [3, 4]. Since 2009, the main Ministry of Health implementation activities relevant for health facility and health workers’ ability to deliver the new case management policy included revision of national malaria case management guidelines, provision of several rounds of in-service training for health workers, distribution of job aids, procurement and supply of anti-malarial medicines, and malaria rapid diagnostic tests (RDT), and implementation of supportive supervision for health workers [5]. The RDT used in Vanuatu is CareStart Combo *Pan Pf* test detecting both *Plasmodium falciparum* and *Plasmodium vivax,* including mixed infections.

Unfortunately, the translation of malaria case management policies and implementation activities into clinical practice recommended by national guidelines cannot be assumed. The findings from high malaria risk countries suggest that stock-outs of ACT and RDTs are common [6–8], the coverage with supportive interventions may be sub-optimal [9] and where commodities do exist inadequate testing of febrile patients, non-adherence to test results, and sub-optimal assessment, counselling and drug dispensing practices compromise effectiveness of malaria case management [10–14]. Very few studies report health system readiness and quality of malaria case management outside of Africa [15, 16] and no reports exist from low malaria risk areas in Western Pacific.

Anecdotal concerns existed in Vanuatu about the quality of outpatient management with major queries raised about malaria testing levels for febrile patients, an important first step in case management process in countries where malaria risk is low and aspirations towards malaria elimination exist. As in many malaria-endemic countries, Vanuatu’s routine information system is inadequate to describe clinical practices. A health facility survey was therefore undertaken to evaluate health system readiness to implement malaria case management and quality of outpatient management for patients presenting with fever.

## Methods

### Survey design and data collection

**Fig. 1:**
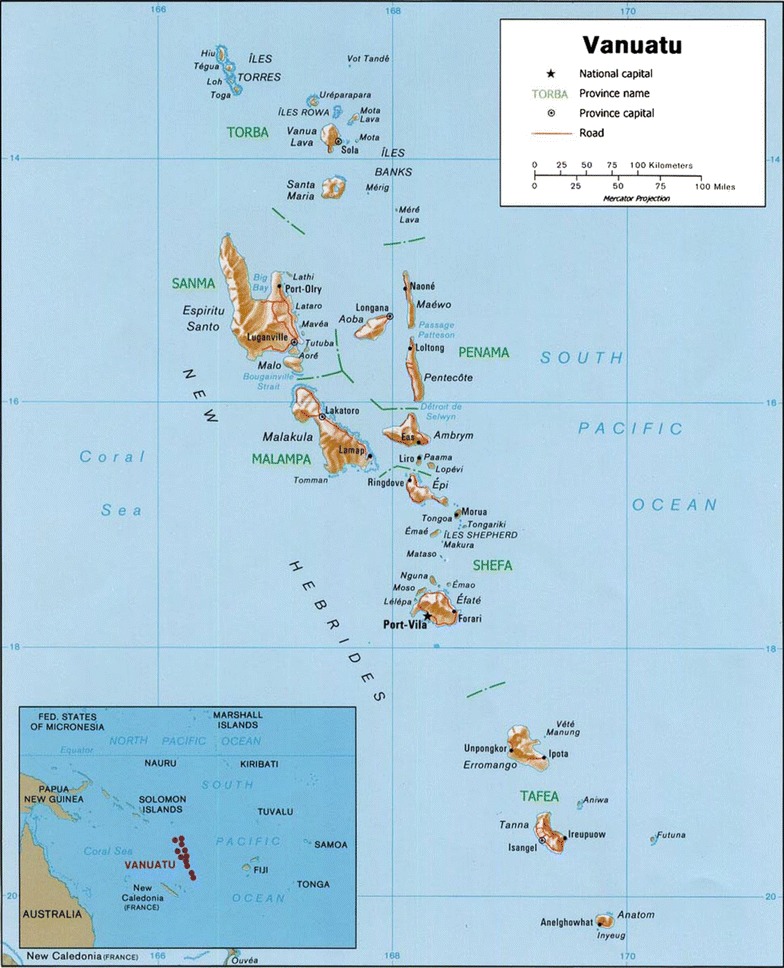
Map of Vanuatu archipelago and its location in Western Pacific

A cross-sectional, cluster sample survey was undertaken between 11 August and 17 September, 2014 during the low malaria transmission season at all functional health centres and hospitals in Vanuatu. Surveyed facilities are located on 17 islands with population of 215,292 inhabitants representing 93 % of all population at risk of malaria in Vanuatu. The map of Vanuatu archipelago is shown in the Fig. 1. The survey did not include private health facilities because there are only 6 clinics countrywide nor widespread network of public aid posts and dispensaries because they are often opened only temporarily, have very low attendance and because treatment-seeking behaviour for malaria is massively skewed towards higher level facilities [2, 17]. It was estimated in 2013 that 57 % of children with fever sought treatment at health facilities [17].

A cluster was defined at health facility level as all initial outpatient visits for patients presenting with fever during working hours (07.30–16.30) over two consecutive working days. Data were collected by six teams, each team composed of two surveyors and supervised by one malaria control officer. The training of data collectors was undertaken over 5 days and included practice with patients and health workers at outpatient departments of the referral hospital in the capital. During the training, the concordance testing based on role plays was undertaken until 90 % agreement of interviewing practice of data collectors in comparison with trainers was achieved.

Data on survey days were collected at each facility using three methods. First, all sick patients, after completing outpatient visit, underwent rapid screening by study teams. The screening included determination of history of fever during the present illness and whether the facility visit was an initial or follow-up. Patients presenting for an initial visit with fever were interviewed, during which information was collected about patient’s age, weight, temperature, previous use of drugs, main complaints reported and history taking and examination practices performed during outpatient consultation, malaria diagnostic tests requested and results reported, medications prescribed and dispensed, and key counselling and drug dispensing tasks performed. Information about the results of the tests, health workers’ diagnoses and medication prescriptions was also collected from prescription slips, laboratory request forms and facility registers where available and appropriate. Second, each facility was assessed for the availability of anti-malarial drugs and RDTs on survey days. The presence of malaria microscopy, weighing scales, thermometers and other basic infrastructure and equipment was also established. Finally, health workers who performed outpatient consultations on survey days were interviewed at the end of the second working day to collect information on their demographics, pre-service training, access to guidelines and job aids, and retrospective exposure to in-service training and supervision. During the interviews their knowledge about malaria treatment policies and interpretation of RDT results was assessed using open-ended questions and RDT images of five possible test outcomes of CareStart Combo *Pan Pf* test.

### Case management recommendations

Health workers’ knowledge and practices were compared to case management recommendations from the national malaria treatment guidelines [3] and health workers’ manual for malaria case management training [4]. The recommended first-line treatment for both *P. falciparum* and *P. vivax* uncomplicated malaria is artemether-lumefantrine (AL). In addition, non-pregnant patients older than 4 years with vivax malaria and no G6PD deficiency should receive a 0.5 mg/kg radical treatment of primaquine for 14 days. Patients with unknown G6PD status should receive half dose of primaquine and either be admitted for monitoring of adverse reactions or as outpatients clinically evaluated on a daily basis for 3 days. The recommended pre-referral treatment for suspected severe malaria is artesunate, administered either as suppository or intramuscularly. Severe malaria should be treated with intravenous artesunate. In special patient groups, AL and intravenous artesunate are respective treatment for uncomplicated and severe malaria in children below 5 kg and in the second and third trimester, while quinine is recommended in the first trimester of pregnancy. Finally, chloroquine, as a weekly prophylaxis, is recommended throughout pregnancy.

With respect to testing and interpretation of test results, the initial guidelines developed in 2009 were ambiguous without defining who should be tested for malaria and allowing anti-malarial treatment for patients with negative test results [3]. These ambiguities were streamlined in the 2012 manual for case management training, which explicitly recommended testing of every patient with fever either with malaria RDTs or malaria microscopy, and subsequent anti-malarial treatment for only test-positive patients [4]. In addition to the recommended performance of assessment tasks across all age groups, the training manuals promoted IMCI approach to the management of children with fever [4]. In summary, an emphasis during the training for health workers has been put on the systematic assessment of patients with fever, which should include history and examination tasks to assess severity of illness, other malaria signs and symptoms, and alternative causes of fever. The recommended history-taking tasks include asking about history of fever, chills, headache, cough, difficult breathing, diarrhoea, vomiting, ear, throat and skin problems, convulsions, ability to drink/breastfeed and vomiting everything. The promoted examination tasks included measuring weight, temperature, pulse, breath count, chest auscultation, checking for neck stiffness, pallor, throat and ear problem, signs of dehydration, abdomen palpation, and looking for altered consciousness, deep breathing, and chest in-drawing [4].

### Analytic approaches and statistical analysis

Data entry and management was undertaken using Access (Microsoft, USA). All forms were entered twice by two independent data-entry clerks and completed data files compared for data-entry errors using verification programs and referring to paper-based questionnaires. The analysis was performed in STATA, version 11 (StataCorp, College Station, TX, USA). Descriptive analysis was undertaken at the health facility, health worker, and patient level. First, to assess health system readiness to implement new case management policy, the analysis was undertaken at the health facility and health worker level. The key indicators at the facility level were proportions of facilities having RDTs in stock, providing functional malaria microscopy and having non-expired anti-malarial drugs in stock. Functional malaria microscopy was defined by the presence of working microscope and microscopist on survey days. The key indicators at health worker level referred to the proportions of health workers trained to perform RDTs, exposed to case management training, having access to malaria treatment guidelines and RDT job aids, having received malaria supervisory visit in past 6 months, and having shown correct knowledge of malaria treatment policies and RDT result interpretations. The knowledge was also stratified by exposure to relevant in-service training. Second, to assess practices at the patient level, the analysis of history and clinical examination assessments, malaria testing and treatment practice was undertaken among all patients with fever. History-taking tasks were defined as performed if patients spontaneously reported symptoms as their main complaints or if health workers asked about their presence when complaints were not reported. The analysis of history and clinical examination tasks was also stratified for patients below and above 5 years of age. The use of medicines was assessed among all patients with fever and in relation to malaria test results and health workers’ routine diagnoses and by age where sufficient sample size allowed. Fourth, to assess the quality of dispensing and counselling practices, the analyses were restricted to patients who had medicines dispensed at the facility. All analyses estimating confidence intervals (CI) around indicators at the patient level were adjusted for clustering at health facility level.

Since the primary study finding of interest for malaria case management implementers was low testing rates, the predictor analysis examining association between health workers’ decision to test for malaria and health facility, health worker and patient level factors was also performed. The logistic regression using generalized estimating equations with an independent working correlation matrix was applied to account for the correlated nature of the data. In the univariate analysis odds ratios (OR), P-values and 95 % CIs were estimated for the association between the outcome and the following predictors: health facility type (hospital vs health centre); availability of malaria microscopy; health workers’ pre-service training (nurse practitioner/doctor vs lower cadres); patients age (≥5 years vs below 5 years), temperature (≥37.5 °C vs lower than 37.5 °C), main complaints of fever, cough, diarrhoea and runny nose, and five health worker factors of programmatic interest for policy implementers (exposure to relevant malaria case management training; RDT training; malaria supervision in past 6 months; treatment guidelines and RDT job aid). Finally, the predictors with *P* value <0.15 were entered into multivariate model. All hypothesis testing and CI estimations were done with an alpha level of 0.05.

### Ethical consideration

During the surveys, all caregivers, adult patients and health workers were provided with a consent information sheet and informed written consent was requested for all participating patients and health workers. The ethical approval for the study was provided by the Ethical Review Committees of the Ministry of Health in Vanuatu (reference number MOH/DG 07/13-VT-tq) and of WHO Western Pacific Regional Office (ID: 2014.17.VAN.2.MVP).

## Results

### Sample description

The survey was undertaken at 41 health facilities. All operational health centres and hospitals in Vanuatu were surveyed. No health worker, adult patient or caregiver of sick child refused to participate in the study. At 41 surveyed facilities interviews were performed with 67 health workers who saw on survey days 892 outpatients, of which 226 (25.3 %) reported fever during the present initial illness. The large majority of health facilities were health centres (35; 85.4 %) and nearly all (40; 97.6 %) belonged to the Ministry of Health. Of 67 health workers who performed outpatient consultations on survey days, 28 (41.8 %) were female, 26 (39.4 %) were in-charge of health facilities and health workers’ median age was 40 years [IQR 35–48]. The large majority (79.1 %) of health workers were nurses (25 registered and 27 nurse practitioners) followed by nurse aids (10.4 %) and midwives (9.0 %). Only one doctor performed outpatient consultations.

Of 226 patients with fever, 111 (49.1 %) were below 5 years of age and 115 (50.9 %) were 5 years and older. The mean age of patients was 14 years. Females represented 45.3 % and among women older than 12 years of age, four women reported pregnancy. The average duration of fever was 3.5 days and half of the patients (49.8 %) sought care to the facility within 48 h. During the facility visit, only 16.6 % of patients had axillary temperature ≥37.5 °C. Fever was most commonly reported as the main complaint (90.3 %), followed by cough (54.9 %), headache (20.8 %), runny nose (15.5 %), joint pain (7.5 %) and diarrhoea (6.6 %). No patients took anti-malarial treatment prior to coming to the facility and 12 (5.3 %) patients were admitted or referred for hospitalization to another facility.

### Health facility and health worker readiness to implement ‘test and treat’ policy

Of basic infrastructure and equipment, nearly all of 41 surveyed facilities had electricity (95.1 %), water (95.1 %), mobile network signal (87.8 %), functional thermometers (100 %), and weighing scales for children (95.1 %) and adults (95.1 %). Similarly, all health facilities had survey day capacity for parasitological diagnosis of malaria either by providing malaria microscopy or by using RDTs (Table 1). Only one health facility did not have RDTs in stock. Malaria microscopy was routinely provided at 63.4 % of facilities. Six facilities, however, either lacked working microscope or microscopy personnel on survey days which resulted in a functional microscopy coverage of 48.8 %. During survey days 95.1 % of health facilities stocked at least one of four weight-specific AL packs, while three-quarters (75.6 %) of facilities were found with all packs in stock. The other recommended anti-malarial medicine, apart from nearly universally available chloroquine (97.6 %), were less commonly found, ranging from 48.8 % for quinine injections to 73.2 % for artesunate suppositories (Table 1).

**Table 1:** Health facility and health worker readiness to implement ‘test and treat’ policy

Health facilities (N = 41)	n	%
Malaria diagnostic services		
Routinely providing malaria microscopy service	26	63.4
Malaria microscopy service functional on survey days	20	48.8
Malaria RDTs in stock	40	97.6
Any functional diagnostics on survey days (microscopy or RDTs)	41	100
Anti-malarial medicines (non-expired)		
Artemether-lumefantrine (at least one pack in stock)	39	95.1
Artemether-lumefantrine (all 4 packs in stock)	31	75.6
Artesunate suppositories	30	73.2
Artesunate injections	27	65.8
Primaquine tablets	26	63.4
Chloroquine tablets	40	97.6
Quinine tablets	26	63.4
Quinine injections	20	48.8

Health workers (N = 67)	n	%
Exposure to in-service training		
Trained how to perform RDTs	47	70.2
Ever trained on malaria case management	40	59.7
Trained on malaria case management since 2009^a^	36	53.7
Trained on malaria case management since 2012^a^	21	31.3
Exposure to job aids		
Malaria treatment guideline	46	68.7
RDT use job aid	47	70.2
Exposure to supportive supervision in past 6 months		
Any supervisory visit	49	73.1
Malaria supervisory visit	45	67.2

^a^All training since 2009 recommended AL and artesunate treatment while only training from 2012 recommended testing of all patients with fever and anti-malarial treatment of only test-positive results. Training prior to 2012 did not provide testing criteria and was ambiguous about interpretation of test-negative results

Health workers’ exposure to in-service case management training and supportive supervision was variable (Table 1). Over two-thirds (70.2 %) of health workers were trained how to perform RDTs, 53.7 % were trained on new malaria treatment policies and 31.3 % were trained on new malaria treatment polices including recommendations to test all patients with fever and treat only test-positive patients. With respect to the supportive supervision, 73.1 % of health workers reported at least one supervisory visit in past 6 months, of which nearly all (91.8 %) were malaria-specific visits. Finally, 68.7 and 70.2 % of health workers had access to respective malaria-specific job aids such as treatment guidelines and RDT use job aid (Table 1).

### Health workers’ knowledge about malaria treatment policies and RDT interpretation

Most health workers (83.6 %) knew the recommended first-line treatment for uncomplicated falciparum malaria, yet their knowledge about treatment recommendations for uncomplicated vivax and mixed infection malaria, pre-referral and severe malaria treatment, and all treatment polices regarding special patient groups, such as those for pregnant women and low-weight children, was sub-optimal (Table 2). Less than half of health workers (47.8 %) knew that artesunate suppository or injections are recommended pre-referral treatment, about quarter (26.9 %) knew of radical primaquine treatment in addition to AL for uncomplicated vivax malaria, and only 22.7 % responded correctly that parenteral artesunate is the treatment of severe malaria. A third of health workers (32.8 %) knew about G6PD testing prior to the first treatment with primaquine. Health workers who attended malaria case management training showed higher knowledge about all treatment policies, however, even in this group of health workers significant knowledge gaps exist (Table 2).

**Table 2:** Health workers’ knowledge about malaria treatment policies and interpretation of RDT results, by training exposure

Knowledge of treatment policies (correct response in brackets)	Trained (N = 36)	Not trained (N = 31)	All HWs (N = 67)
	n (%)	n (%)	n (%)
First-line treatment policy for uncomplicated falciparum malaria			
Adults and children above 5 kg [AL]	32 (88.9)	24 (77.4)	56 (83.6)
Children below 5 kg [AL]	22 (61.1)	17 (54.8)	39 (58.2)
Pregnant women in first trimester [QN]	10 (27.8)	2 (6.5)	12 (17.9)
Pregnant women in second & third trimester [AL]	21 (58.3)	15 (48.4)	36 (53.7)
First-line treatment policy for uncomplicated vivax malaria			
Adults and children above 4 years [AL + PQ]	15 (41.7)	3 (9.7)	18 (26.9)
Children below 4 years [AL]^a^	21 (58.3)	15 (50.0)	36 (54.6)
Pregnant women in first trimester [QN]	7 (19.4)	2 (6.5)	9 (13.4)
Pregnant women in second and third trimester [AL]	17 (47.2)	11 (35.5)	28 (41.8)
First-line treatment policy for uncomplicated mixed infection (including *P. falciparum*) malaria			
Adults and children above 4 years [AL + PQ]	9 (25.0)	2 (6.5)	11 (16.4)
Children below 4 years [AL]	22 (61.1)	14 (45.2)	36 (53.7)
Pregnant women in first trimester [QN]	9 (25.0)	2 (6.5)	11 (16.4)
Pregnant women in second & third trimester [AL]	18 (50.0)	10 (32.3)	28 (41.8)
Pre-referral Rx for suspected severe malaria [AS sup/inj]	21 (58.3)	11 (35.5)	32 (47.8)
First-line treatment policy for severe falciparum malaria			
Adults and children above 5 kg [AS inj]^a^	11 (31.4)	4 (12.9)	15 (22.7)
Children below 5 kg [AS inj]^a^	5 (14.3)	3 (9.7)	8 (12.1)
Pregnant women in first trimester [QN inj]^b^	8 (22.9)	4 (13.3)	12 (18.5)
Pregnant women in second & third trimester [AS inj]^b^	5 (14.3)	4 (13.3)	9 (13.9)

Knowledge of *Carestart Pan Pf* results interpretation	Trained (N = 45)	Not trained (N = 20)	All HWs (N = 65)^b^
	n (%)	n (%)	n (%)
Positive *P. falciparum* result	36 (80.0)	8 (40.0)	44 (67.7)
Positive non-*P. falciparum* (*P. vivax/P. malariae*) result^a^	31 (68.9)	9 (45.0)	40 (61.5)
Positive mixed infection result	38 (84.4)	10 (50.0)	48 (73.9)
Negative result	37 (82.2)	11 (55.0)	48 (73.8)
Invalid result	29 (64.4)	8 (40.0)	37 (56.9)
All five test readings correct	25 (55.6)	6 (30.0)	31 (47.7)
All three test positive readings correct	31 (69.0)	7 (35.0)	38 (58.5)

^a^One health worker excluded due to missing response values

^b^Two health workers excluded due to missing response values

With respect to health workers’ knowledge about interpretation of *Carestart Combo Pan Pf* RDT results, less than half of health workers (47.7 %) gave correct responses to all five test outcomes, while correct reading of individual test results ranged between 56.9 and 67.7 %. Health workers who were trained on RDT use showed better knowledge compared to untrained health workers, yet in the trained group the knowledge of all five readings has not reached more than 55.6 % (Table 2).

### Health workers’ assessment practices

**Fig. 2:**
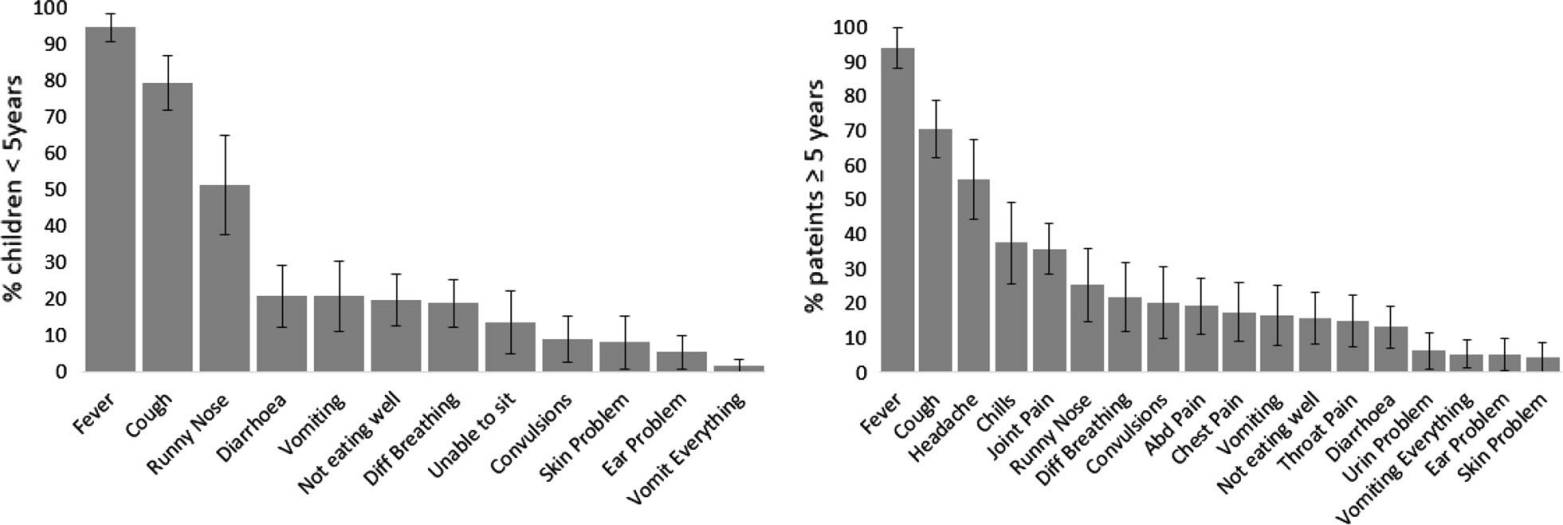
Health workers’ history-taking practices for patients with fever, by age

Of basic assessment tasks for 226 patients with fever, the large majority of patients (87.1 %) were asked for their age while weight and temperature was taken for 31.2 and 46.4 % of patients, respectively. Previous use of medicines during the present illness was asked for 27.6 % of patients. Health workers’ history-taking practices are presented by age in Fig. 2. Across all age groups, assessment of fever (94.2 %) and cough (74.8 %) was performed for the majority of patients, less than half (38.1 %) had runny nose assessed, while the other history-taking tasks referring to alternative causes of fever, such as throat (7.5 %), ear (5.3 %), urinary (4.4 %), skin (6.2 %), and other problems, were rarely performed. Among children below 5 years of age, danger signs such as history of convulsions (9.0 %), ability of drinking or breastfeeding (13.8 %), and vomiting everything (1.8 %), were rarely asked about (Fig. 2). The symptoms with higher assessment levels were those that were more commonly spontaneously reported by patients as their main complaints (90.3 and 54.9 % for fever and cough, respectively).

**Fig. 3:**
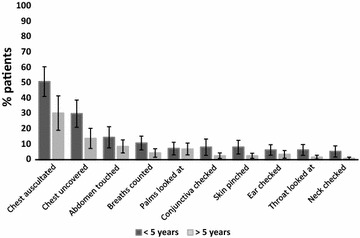
Health workers’ clinical examination practices, by age

Clinical examination practices for patients with fever stratified by age are presented in Fig. 3. Across all age groups the only task performed for a substantial proportion of patients was chest auscultation (40.4 %) while all other tasks were performed for less than one-fifth of the patients. Of 69 children below 5 years of age presenting with the complaint of cough or difficult breathing, the most common task performed was chest auscultation (62.3 %) while uncovering of chest and breath count was, respectively, done for 37.7 and 13.0 % of children. Finally, of 14 children presenting with diarrhoea only four (28.6 %) had skin pinched to assess for dehydration.

### Malaria testing and adherence to test result

Of 226 patients presenting with fever, 35.0 % (95 % CI 23.4–48.6) were tested for malaria either by malaria microscopy or RDTs. One patient was tested by both diagnostic methods. Of 79 patients tested (and 80 malaria tests performed) all test results were negative for malaria. Only one patient, who tested negative on microscopy, was treated for malaria with AL and was admitted at the health facility (Table 3).

**Table 3:** Malaria testing and adherence to test result

	n	%	95 % CI
Patients with fever (N = 226)			
Tested for malaria	79	35.0	23.4–48.6
Tested by malaria microscopy	31	13.7	6.4–27.1
Tested by malaria RDT	49	21.7	11.8–36.4
Patients tested for malaria (N = 79)			
Test-positive	0	na	na
Treated for malaria	1	1.3	0–3.8

Fourteen predictors that may have influenced testing for malaria were examined. Table 4 shows the multivariate model and univariate results for programmatic interventions of interest that did not meet the criteria for multivariate analysis (p-value < 0.15). Patients were significantly more likely to be tested if they were older than 5 years (OR = 2.29; 95 % CI 1.23–4.27), seen by less qualified health workers (OR = 2.40; 95 % CI 1.29-4.50), trained to test all patients with fever (OR = 2.39; 95 % CI 1.28–4.47) and if they presented with increased temperature (OR = 2.99; 95 % CI 1.33–6.71), main complaint of fever (OR = 5.34; 95 % CI 1.12–25.45) and without runny nose (OR = 3.49; 95 % CI 1.24–9.84). No significant association was found between testing and programmatic interventions other than training (Table 4). Similarly, no association was found with other examined factors such as health facility type (OR = 1.07; 95 % CI 0.55–2.06), availability of microscopy (OR = 1.47; 95 % CI 0.84–2.55), and patients’ main complaints of cough (OR = 0.72; 95 % CI 0.41–1.25) and diarrhoea (OR = 1.27; 95 % CI 0.43–3.70).

**Table 4:** Predictors influencing performance of malaria test for patients with fever

Predictors	No. patients	No. tested	OR (95 % CI)	P-value
*Multivariate results*				
Health workers’ pre-service training				
Nurse/nurse aid/other	111	50 (45.1)	2.40 (1.29–4.50)	0.006
Doctor/nurse practitioner	113	28 (24.8)	Ref	
Health worker received malaria case management training^a^				
Trained	96	46 (47.9)	2.39 (1.28–4.47)	0.006
Not trained	128	22 (35.0)	Ref	
Patient’s age				
5 years and older	114	49 (43.0)	2.29 (1.23–4.27)	0.009
Below 5 years	110	29 (26.4)	Ref	
Temperature ≥37.5 °C				
Yes	37	19 (51.4)	2.99 (1.33–6.71)	0.008
No	186	59 (31.7)	Ref	
Fever main complaint				
Yes	202	76 (37.6)	5.34 (1.12–25.45)	0.036
No	22	2 (9.1)	Ref	
Running nose main complaint				
Absent	189	72 (38.1)	3.49 (1.24–9.84)	0.018
Present	35	6 (17.1)	Ref	
*Univariate results* ^b^				
Health worker received malaria supervision in past 6 months				
Yes	157	55 (35.0)	1.03 (0.57–1.88)	0.919
No	67	23 (34.3)	Ref	
Health worker has access to malaria treatment guideline				
Yes	172	64 (37.2)	1.61 (0.81–3.20)	0.175
No	52	14 (26.9)	Ref	
Health worker trained how to perform RDTs				
Yes	181	63 (34.8)	1.00 (0.50–2.00)	0.992
No	43	15 (34.9)	Ref	
Health worker has access to RDT job aide				
Yes	164	57 (34.8)	0.99 (0.53–1.84)	0.973
No	60	21 (35.0)	Ref	

^a^Only relevant case management training recommending testing of all fevers for malaria

^b^Selected predictors of programmatic interest

### Diagnosing, treatment, dispensing, and counselling practices

Health workers made 255 diagnoses for 226 patients or, on average, 1.1 diagnoses per patient. No diagnosis of malaria was made. The most common diagnoses made by health workers were cough/cold (61; 27.0 %), upper respiratory tract infection (URTI) (58; 25.7 %), influenza (21; 9.3 %), fever (17; 7.5 %), back/body pain (12; 5.3 %), gastroenteritis (8; 3.5 %), pneumonia (8; 3.5 %), and skin infection (8; 3.5 %). No diagnosis was made for seven (3.1 %) of patients. Health workers prescribed 467 medicines or, on average, 2.1 drugs per patient. The large majority of patients were treated with antipyretics (187; 82.7 %) and antibiotics (175; 77.4 %). All of eight patients diagnosed with pneumonia were treated with antibiotics but also the majority of patients with diagnosis of URTI (90 %), influenza (81 %), cough/cold (79 %), ‘fever’ (72 %), and diarrhoea/gastroenteritis (53 %).

Of 175 patients prescribed antibiotics during the outpatient visit, 161 patients were sent home with dispensed oral antibiotics. The majority of these patients (94.4 %) were explained how to take tablets at home and 68.5 % were told to complete all doses. Antibiotics were however prescribed and dispensed based on weight for 27.6 % of patients, the first dose was given at facility for only 4.9 % of patients and advice on what to do in case of vomiting was rarely provided (1.2 %). Less than one-quarter of patients were advised to return immediately if they become sicker (22.4 %) or in few days if fever persists (18.6 %). No significant differences were observed in dispensing and counselling practices between young children and patients 5 years and older (Table 5).

**Table 5:** Dispensing and counselling practices for febrile patients treated with antibiotics

Dispensing and counselling tasks	<5 years (N = 81)			5 years and older (N = 81)			All age groups (N = 162)		
	n	%	95 % CI	n	%	95 % CI	n	%	95 % CI
Explained how to take drugs	76	93.8	87.3–97.1	77	95.1	85.5–98.4	153	94.4	90.1–97.0
Advised to complete all doses	52	64.2	49.5–76.7	59	72.8	51.7–87.1	111	68.5	52.6–81.1
Weighed	27	33.3	18.1–53.1	19	24.1	9.6–48.5	46	28.8	15.2–47.6
Asked to return immediately if sicker^a^	16	19.8	10.7–33.5	20	25.0	14.7–39.1	36	22.4	14.9–32.2
Asked to return if fever persists^a^	13	16.1	8.6–27.9	17	21.3	12.6–33.5	30	18.6	12.3–27.2
First dose given at HF	6	7.4	2.6–19.5	2	2.5	0.3–17.3	8	4.9	1.3–16.6
Advised what to do if vomiting	1	1.2	0.2–9.6	1	1.2	0.2–9.6	2	1.2	0.3–5.5

^a^Denominator does not include one patient with a missing value response

## Discussion

Health system readiness and outpatient management of febrile patients 5 years following scale-up of ‘test and treat’ policy for malaria was evaluated in Vanuatu. The findings revealed several successes but also gaps that need to be addressed by policy implementers to optimize the quality of outpatient service delivery.

The availability of RDTs and AL at health facilities, as the basic prerequisite for implementation of ‘test and treat’ policy for uncomplicated falciparum malaria, was very high. In rare cases where RDTs were out of stock, malaria microscopy was provided and therefore the capacity for parasitological diagnosis of malaria was ensured universally. High availability of RDTs and ACT found in Vanuatu is in contrast with common stock-out reports of the same items found in many high malaria risk countries in Africa [6–8]. Yet in Vanuatu, where *P. vivax* is common and indeed predominant over *P. falciparum* [18], it is worrisome that over a third of facilities were found without any stock of primaquine. Similar findings on lower availability of primaquine for radical *P. vivax* treatment was also reported from other countries harbouring both *P. falciparum* and *P. vivax* transmission [15, 16]. By mid-2014 the coverage of health workers trained on malaria case management, how to perform RDTs, and exposed to job aids and supportive supervision was not yet optimal in Vanuatu, ranging from 50 to 70 % between indicators, but was, however, higher compared to the coverage reported from other countries where national evaluations were performed after a similar time period since the beginning of ‘test and treat’ policy implementation [9, 19].

Health workers’ knowledge about malaria treatment policies was limited to relatively high awareness of the first-line policy for uncomplicated falciparum malaria (84 %) while knowledge about treatment recommendations for *P. vivax,* mixed infections, pre-referral and severe malaria treatment, and about polices with respect to special patient groups was significantly lower. Given the scarcity of severe cases in Vanuatu [20, 21] and uncommon exposure in practice, the sub-optimal knowledge about policies for severe malaria, including special patient groups across spectrum of disease severity, was less surprising. It was, however, more surprising to observe that the knowledge about radical primaquine treatment for *P. vivax* was very low despite a long-term presence of the policy at health centres and hospitals and exposure to in-service training. Similarly, it was worrisome to observe that only about half of health workers were able to correctly interpret all RDT result outcomes while misclassification of test positivity and species was common despite the training provided.

With respect to clinical practices, sub-optimal history taking and clinical examination practices were found. Assessments were often limited to patients’ spontaneous reports of the main complaints (most commonly fever and cough) with little systematic search for other signs and symptoms. Despite the universal availability of malaria diagnostics, only 35 % of patients with fever were tested for malaria, the rates generally lower compared to what was observed in countries with higher malaria transmission following implementation of testing policy [9, 11–13, 15]. Several factors influencing testing practices were revealed. First, patients seen by nurses and nurse aids were more likely to have malaria test done compared to more qualified health workers. A possible explanation may be that nurse practitioners and doctors have greater knowledge and clinical skills and therefore ignorance or perception of being superior to guidelines may result in lower systematic testing of febrile patients among these health workers. Higher adherence to malaria guidelines by lower cadres of health workers is not unusual and has been reported previously [22, 23]. Second, older children and adults were more likely to be tested compared to children below 5 years of age which may be a reflection of old policies allowing presumptive treatment in young children, or simply due to greater simplicity in performing malaria testing in older children and adults. Third, the patients presenting with increased temperature, main complaint of fever and absence of runny nose were also more likely to be tested for malaria. The findings suggest health workers’ intention to rule out malaria based on more specific clinical signs and symptoms—the practice understandable in low transmission area, however, at the cost of reduced sensitivity [24, 25] and in discordance with Vanuatu policy promoting widespread use of diagnostics, high suspicion of malaria and minimization of missed cases on path to elimination where each and every case should count. Finally, with respect to key programmatic interventions, patients seen by health workers trained to test all fevers for malaria were more likely to perform the test, however, no significant association was found between malaria testing and supportive supervision. These findings do not discard supportive supervision as potentially effective intervention but do raise questions about the quality and modalities of this intervention the way it was implemented in Vanuatu, by clinically non-qualified personnel focused on record reviews rather than on observing and supporting health workers to adhere to national guidelines.

More positively, when health workers do test for malaria non-adherence to test negative results is negligible, with probably only 1 % of such patients receiving anti-malarial treatment. Although improving trends in adherence to test negative results have been reported [9] these findings are however in contrast with reports from other malaria-endemic countries [9, 26] including those outside of Africa [27]. Adherence to test results, and primarily to negative ones, dependent on accumulated experience with test-positive results has been suggested as one of the major drivers of the ‘test and treat’ policy adoption [28]. In Vanuatu, health workers do not see many test-positive results and what remains unclear is whether, and on what scale, non-adherence existed in past and whether, hopefully, future increase in testing would preserve the same adherence levels as shown in this study. Unfortunately, very high use of antibiotics for patients with fever (77 %) was found. Moreover, it was striking that the large majority of patients with diagnoses highly suggestive of viral diseases such as URTI, cough/cold, influenza, and diarrhoea/gastroenteritis are treated with antibiotics. Among rare reports about drug use in Vanuatu it was found that the rational drug use training for health workers was launched in 1988 when the use of antibiotics for cough and cold was unacceptably high [29]. Over 25 years later, this irrational practice, which may have been facilitated by the decline in malaria transmission, has obviously persisted. Irrational drug use accompanied by sub-optimal dispensing and counselling practices, as shown in this study, severely threatens efficacy and effectiveness of antibiotic treatment in Vanuatu.

Since malaria is becoming an uncommon disease in Vanuatu it may easily be omitted (or even forgotten) as diagnosis consideration by health workers. Standalone interventions, such as in-service training, may temporarily raise awareness of malaria, however, ongoing interventions are needed to keep malaria on health workers’ radar and optimize clinical practices long term. The supportive supervision is one of such quality improvement interventions that is generally shown to be effective in improving health workers’ performance and is indeed implemented in Vanuatu, albeit with sub-optimal quantitative and qualitative coverage. The effective supportive supervision requires adequate human and financial resources, and in a country such as Vanuatu, with shortage of clinical personnel, difficult and expensive physical access to peripheral facilities, significant further investments are required to implement cost-effective supervision. Vanuatu has, however, resolved communication problems through optimum mobile phone network and ownership of phones, and in such settings interventions using, e.g., regular text-message information sent to health workers [30, 31], may complement existing interventions and present another strategy to provide ongoing support to health workers to re-inforce malaria testing and a minimum set of clinical standards. Yet, whichever interventions are implemented, appropriate management of non-malaria febrile illnesses and rational use of antibiotics should be an integral part of the quality improvement.

Several study limitations should be mentioned. First, testing rates may have been higher and adherence to test-negative results lower if the survey was undertaken during high malaria transmission season. Second, non-inclusion of small health facilities limits generalizability of results to health centres and hospitals. Third, assessment and counselling data collected through exit interviews have likely minimized Hawthorn effect (health workers performing better than usual when observed) but may have also been subject to recall and courtesy biases through the responses. Fourth, the study focus was on adherence to guidelines from health workers’ perspective while the impact of practices or guidelines on ‘true’ malaria or the accuracy of routine malaria test results was not established. Similarly, the use of antibiotics was not assessed based on needs for antibiotics but for all patients with fever and according to the routine health workers’ diagnoses. This limitation was however unlikely to change the conclusion about irrational antibiotic use given the massive use observed.

## Conclusions

Five years following scale-up of ‘test and treat’ policy in Vanuatu, health facilities are better equipped to implement policy for uncomplicated falciparum than vivax malaria, health workers are moderately exposed to support interventions, and health workers’ knowledge about interpretation of RDT results and treatment recommendations for other than falciparum malaria policy is rather low. History-taking and clinical examination practices for patients with fever are sub-optimal. Malaria testing rates for febrile patients are low, however, when tests are performed patients with negative-test results are rarely treated with anti-malarial medicines. Spontaneously reported symptoms are largely equalized with antibiotic use resulting in their massive and irrational use. Health workers’ decision to test appears to be influenced by clinical judgements overruling recommendations. Investments into quality improvements of ongoing interventions, such as supportive supervision, innovative quality improvement strategies, such as mobile phone communication with health workers, and regular assessments of case management quality are needed to monitor and re-inforce clinical practices in this archipelago area characterized by remoteness and shortage of personnel but aspiring towards malaria elimination.
